# Climate variability reduces biomass stability in species-rich grasslands, depending on management

**DOI:** 10.1007/s00442-026-05925-2

**Published:** 2026-07-16

**Authors:** Yva Herion, Martin Andrzejak, Harald Auge, Walter Durka, Sylvia Haider, Johannes Höfner, Lotte Korell, Anna-Maria Madaj, Stefan Michalski, Erik Welk, Anke Hildebrandt, W. Stanley Harpole, Christiane Roscher

**Affiliations:** 1 Department of Physiological Diversity, Helmholtz-Centre for Environmental Research (UFZ), Permoserstraße 15, 04318 Leipzig, Germany; 2 German Centre for Integrative Biodiversity Research (iDiv) Halle-Jena-Leipzig, Puschstraße 4, 04103 Leipzig, Germany; 3 Department of Community Ecology, Helmholtz-Centre for Environmental Research (UFZ), Theodor-Lieser-Str. 4, 06120 Halle (Saale), Germany; 4 Faculty of Natural Sciences 1 – Biosciences, Martin-Luther-University Halle-Wittenberg (MLU), Am Kirchtor 1, 06108 Halle (Saale), Germany; 5 Ecology and Genetics, University of Oulu, Pentti Kaiteran katu 1, 90570 Oulu, Finland; 6 Institute of Ecology, School of Sustainability, Leuphana University of Lüneburg, Universitätsallee 1, 21335 Lüneburg, Germany; 7 Department of Species Interaction Ecology, Helmholtz-Centre for Environmental Research (UFZ), Permoserstraße 15, 04318 Leipzig, Germany; 8 Institute of Biology, Leipzig University, Talstraße 33, 04103 Leipzig, Germany; 9 Department of Computational Hydrology, Helmholtz-Centre for Environmental Research (UFZ), Permoserstraße 15, 04318 Leipzig, Germany; 10 Institute of Geoscience, Friedrich-Schiller-University Jena, Burgweg 11, 07749 Jena, Germany

**Keywords:** Climate change experiment, Drought, Recovery, Resistance, Stabilizing effects

## Abstract

Climate change threatens grassland biomass stability, but combined effects of climate variability and grassland management have rarely been analyzed, constraining our ability to predict management-dependent responses. In a large field experiment in Central Germany, we assessed temporal stability of aboveground biomass and potential underlying mechanisms (dominance, asynchrony, and averaging) in non-fertilized species-rich grasslands over nine years (2015–2023). The grasslands were exposed to ambient or simulated future climate following regional projections (higher temperature and altered seasonal precipitation pattern) and were managed by mowing or sheep grazing. While the future climate treatment showed little effects, high background climate variability increased biomass variability due to lower stabilization by asynchrony and averaging, which was more pronounced in meadows than in pastures. The lower biomass resistance to extreme summer droughts and the higher recovery afterwards in meadows compared to pastures was likely attributable to higher amounts of biomass removed by mowing than by grazing. However, stabilization by the diversity-associated averaging effect, which generally contributed most to biomass stabilization, was lower in pastures compared to meadows at peak biomass production in spring, due to declining species diversity with the lower biomass removal by grazing. Accordingly, we conclude that grassland management with low biomass removal can enhance biomass resistance to climate variability, but this may be offset in the long term by species loss and reduced stabilization through the diversity-associated averaging effect.

**Supplementary Information:**

The online version contains supplementary material available at 10.1007/s00442-026-05925-2.

## Introduction

Grasslands cover about 25% of the global land area (FAO 2022), and stability of their biomass production is essential for the reliable provision of multiple ecosystem functions and services such as soil fertility, carbon sequestration, and forage production (Isbell et al. 2017; Bengtsson et al. 2019). A key aspect of stability is the temporal variability of biomass, including its resistance to and recovery from environmental perturbations such as extreme climate events (Pimm 1984; Grimm and Wissel 1997). Climate change poses major threats to the stability of grassland biomass by affecting species dynamics within communities and impairing their buffering capacity against environmental fluctuations (Gonzalez and Loreau 2009; Valencia et al. 2020; Luo et al. 2025). Grassland management can offer opportunities to mitigate negative impacts of climate change, but few studies have analyzed the combined effects of climate change and grassland management on biomass stability (e.g., Vogel et al. 2012; Deléglise et al. 2015; Han et al. 2023).

**Fig. 1:**
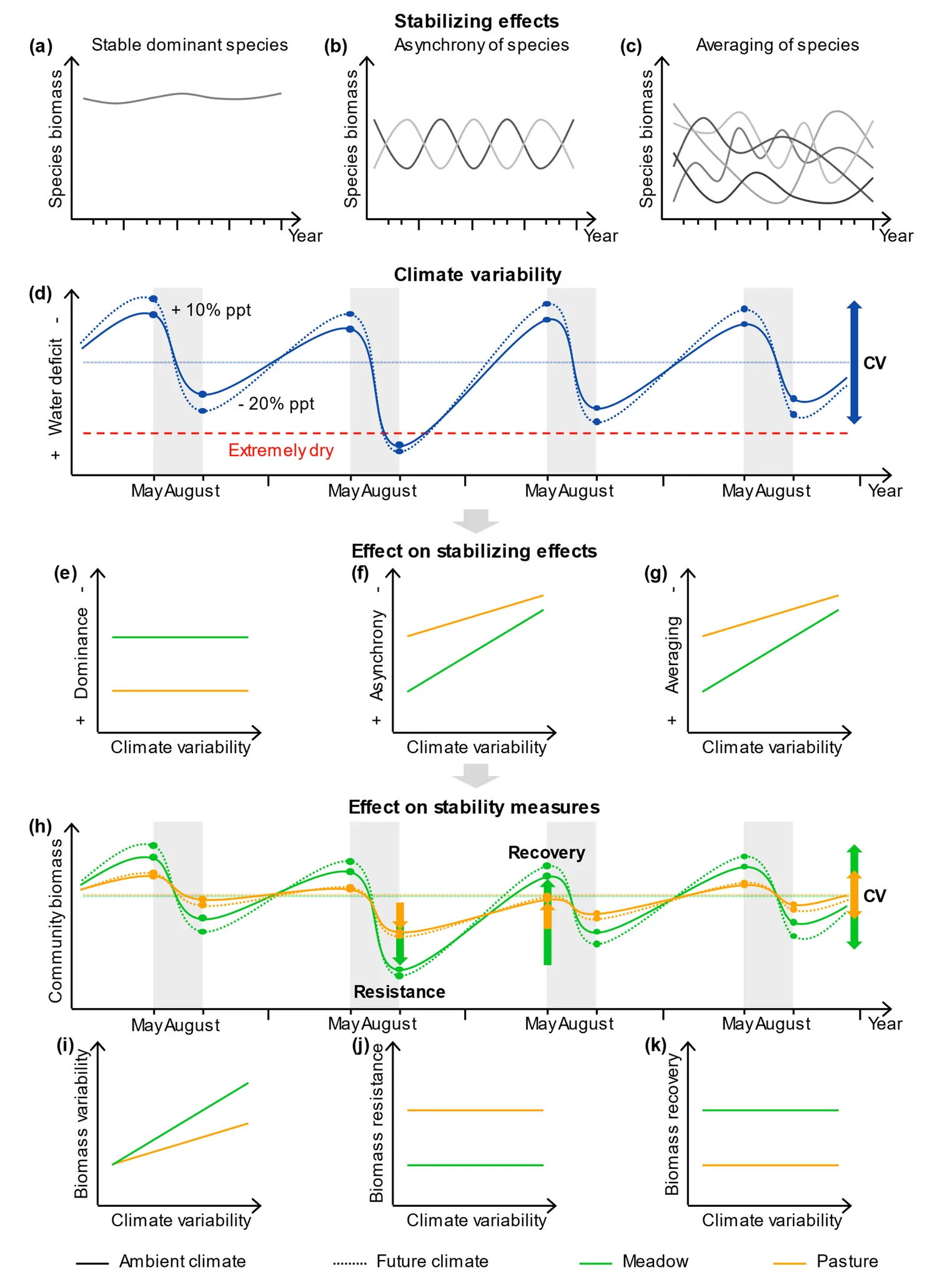
(**a**-**c**) Theoretical biomass fluctuation of different species (gray lines) stabilizing community biomass. (**d**) Expected variability of water deficit under ambient and future climate treatment throughout four exemplary years with high background climate variability including an extreme drought. (**e**-**g**) Hypothesized effects of climate variability on stabilizing effects. (**h**) Expected response of community biomass to climate variability. (**i**-**k**) Hypothesized effects of climate variability on biomass variability, resistance to and recovery from extreme droughts. In case of resistance and recovery, climate variability only refers to the climate treatments and not to background climate. ppt = precipitation, CV = coefficient of variation

Species dynamics within plant communities can stabilize their biomass production. Three different stabilizing effects can be distinguished: stable dominant species (Fig. 1a), species asynchrony (Fig. 1b), and species averaging (Fig. 1c, Tilman 1996; Doak et al. 1998; Grime 1998). Dominant species can account for a large proportion of community biomass and are often found to be more stable than subordinate species (e.g., Roscher et al. 2011; Ma et al. 2017; Valerio et al. 2022). Asynchronous species fluctuations are assumed to be caused by ecological processes such as species-specific responses to environmental variation and interspecific competition (Tilman 1996; Gonzalez and Loreau 2009). A higher species diversity increases the chance that the community includes species which respond differently to environmental fluctuations (insurance hypothesis; Yachi and Loreau 1999). Besides, at any deviation from perfect synchrony, greater species diversity is associated with enhanced statistical averaging (Doak et al. 1998; Segrestin et al. 2024). Studies often did not distinguish between species asynchrony and averaging. However, separating these stabilizing effects enables disentangling the contributions of ecological versus statistical mechanisms to community biomass stability, thereby helping to solve the long-standing debate on the causes of diversity-stability relationships (Doak et al. 1998; Tilman et al. 1998).

Besides the global rise in mean annual temperature, Central Europe is projected to experience rather small changes in total annual precipitation but a shift in seasonality with more precipitation in spring and fall and less in summer (Jacob et al. 2014; IPCC 2023). In observational studies, higher precipitation variability was found to have no or positive effects on the stability of dominant species and either increased or decreased asynchronous species fluctuations (e.g., Hallett et al. 2014; Zhang et al. 2022a, b). Moderate climate variability may promote asynchronous species fluctuations and species diversity, while high climate variability may have negative effects (Gilbert et al. 2020). In some studies, higher precipitation variability reduced temporal stability of grassland biomass (e.g., Zhang et al. 2018; Gilbert et al. 2020), but in others it had no effect (Hallett et al. 2014; Valencia et al. 2020). These differing results from observational studies highlight the current uncertainty regarding the effects of increasing climate variability on grassland biomass stability and point to the potential value of experimental approaches in establishing causality.

Grasslands can harbor a high diversity of plant species (Wilson et al. 2012). Recent studies identified species averaging as the most important stabilizing effect in diverse grasslands (Lisner et al. 2024; Segrestin et al. 2024). In Central Europe, most grasslands have been created by human activities and require regular management to prevent succession to woody systems (Hejcman et al. 2013). Mowing and grazing, two widespread management types, may drive different species dynamics within grassland communities depending on their frequency, intensity, and timing (e.g., Tälle et al. 2016). Low-intensity grazing with a low amount of removed biomass and selective feeding behavior of grazing animals may increase light competition and promote the dominance of tall-growing and less palatable species, which can result in a loss of species diversity over time and less asynchronous species fluctuations (Koerner et al. 2018; Eskelinen et al. 2022; Han et al. 2023). Mowing, in contrast, removes biomass non-selectively and thereby reduces light competition, which can decrease dominance, increase species diversity, and promote asynchronous species fluctuations when applied at moderate frequency (Tälle et al. 2016; Zhao et al. 2024). However, whether management-induced changes in dominance structure and species diversity alter grassland biomass stability is poorly understood (Zhang et al. 2016; Zhao et al. 2024). Independent of the management type, a higher biomass removal through management may increase community biomass variability (quantified by coefficient of variation) due to lower mean biomass or higher drought sensitivity. Low vegetation height and plant cover may decrease the drought resistance of grasslands due to higher soil surface evaporation, further decreasing soil moisture (Vogel et al. 2012). Grasslands that lost more biomass under drought may respond to drought release with an increased post-drought biomass production outperforming less drought-sensitive grasslands (Matos et al. 2020; Hahn et al. 2021). The question is whether the intensity of biomass removal is more important for buffering grassland biomass against climate variability than management-dependent species dynamics within the communities.

**Fig. 2:**
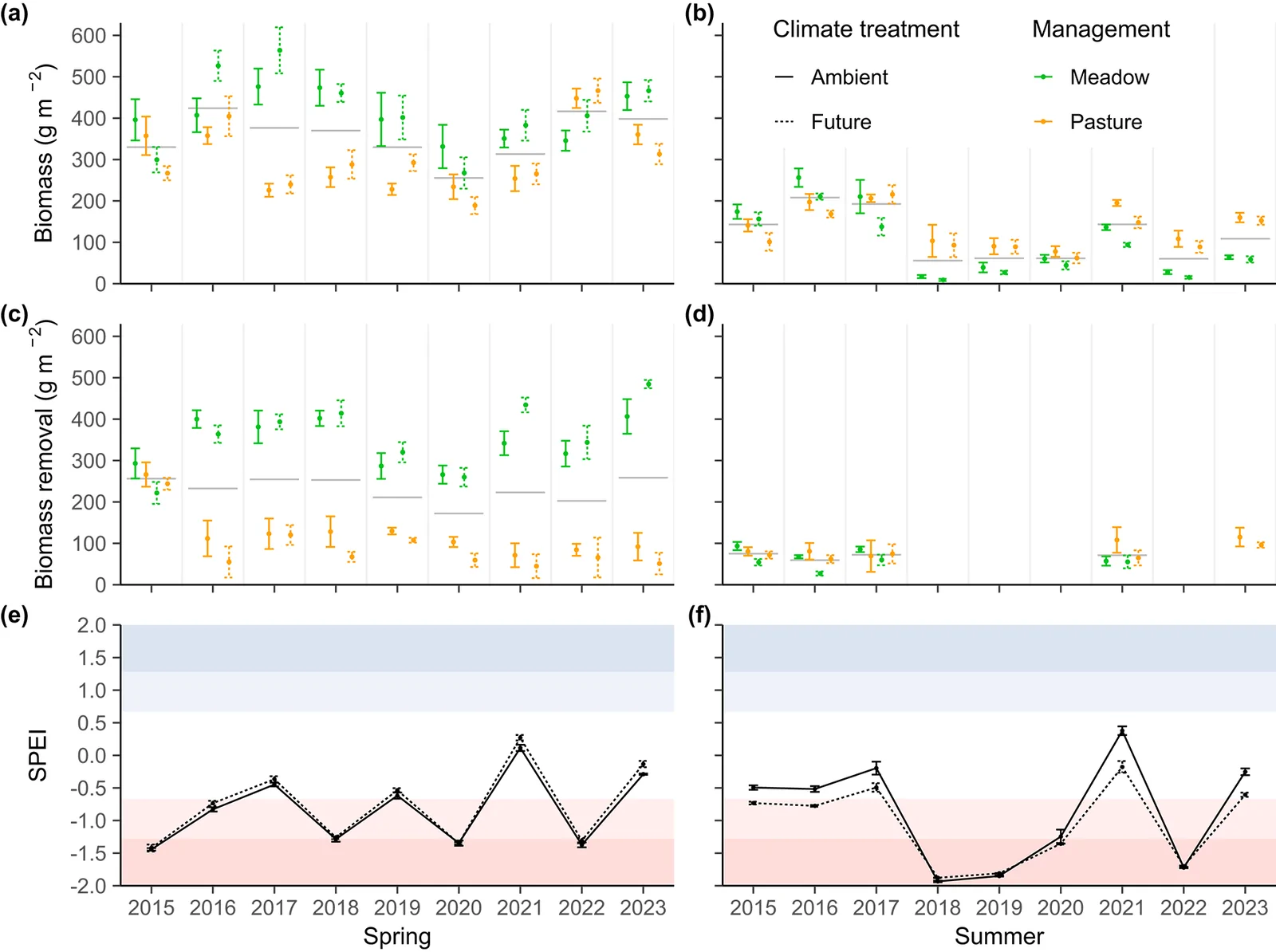
(**a**, **b**) Community biomass of spring (mostly early June) and summer (mostly August) harvests per year and climate × grassland management treatment combination (means ± SE, *n* = 5). (**c**, **d**) Community biomass removal through mowing and grazing directly after spring and summer harvests per year and climate × grassland management treatment combination (means ± SE, *n* = 5). Horizontal gray lines in (**a**-**d**) mark the mean value across all treatment combinations. (**e**, **f**): SPEI (Standardized Precipitation-Evapotranspiration Index) determined by aggregating water deficit over three months (spring: March – May, summer: June – August) and taking into account the last 30 years (1994–2023). The white bands mark normal conditions, the blue bands indicate moderate or extreme wet and the red bands moderate or extreme dry conditions, following Isbell et al. (2015)

In this study, we analyzed a nine-year time series (2015–2023) of aboveground biomass data from species-rich extensively managed meadows and pastures of a large climate change field experiment in Central Germany (Global Change Experimental Facility (GCEF), Schädler et al. 2019). The future climate treatment follows a realistic regional scenario with higher mean temperatures and higher amounts of precipitation in spring and fall but lower amounts of precipitation in summer compared to ambient climate. Both ambient and future climate treatment retain the inter-annual background climate variability, which was high during the study period and included some extreme droughts. The meadows and pastures were initially sown using the same set of plant species and differ only in whether they are mown at moderate frequency or short-term grazed by sheep. Extending a previous study in the same experiment (Korell et al. 2024), we further explore the biomass stability of the meadows and pastures by considering different measures of stability (temporal variability, resistance to, and recovery from droughts), identifying underlying stabilizing effects, and analyzing season-specific effects of experimental future climate and background climate variability. Specifically, we tested the following hypotheses: (1) Higher temporal variability of climate, i.e., stronger seasonal fluctuations under experimental future climate or higher background climate variability (Fig. 1d), results in unchanged stabilization by dominance but lower stabilization by asynchrony and averaging (Fig. 1e-g) and accordingly in higher temporal variability of biomass (Fig. 1h-i), (2) the meadows respond to higher temporal variability of climate with a stronger decrease in stabilization (Fig. 1f-g) and a stronger increase in temporal variability of biomass (lower resistance to but higher recovery from droughts, Fig. 1h-k) than the pastures, because more biomass is removed by mowing than by grazing in our experiment, and (3) in our high-diversity grasslands, species averaging contributes most to stabilization of community biomass in both management types, but stabilization by dominance is lower and stabilization by asynchrony and averaging is higher in meadows than in pastures (Fig. 1e-g).

## Materials and methods

### Experimental site

This study was conducted in the Global Change Experimental Facility (GCEF, Schädler et al. 2019) located at the field research station of the Helmholtz-Centre for Environmental Research (UFZ) in Bad Lauchstädt, Germany (51°23’33’’ N 11°52’59’’ E, 118 m a.s.l). The area is characterized by a temperate, sub-continental climate with mean annual temperature of 10.2 °C and mean annual precipitation of 491 mm over the last 30 years (Gründling et al. 2022). During the study period (2015–2023), mean annual temperature was 10.9 °C and mean annual precipitation 415 mm. The soil of the experimental site is a Haplic Chernozem (Altermann et al. 2005). The GCEF follows a randomized split-plot design with climate as the main-plot factor (ambient and future climate treatment, each replicated in five main plots) and land use as the subplot factor (five subplots of 16 × 24 m per main plot). The future climate treatment (initiated in 2014) was intended to raise daily mean air temperature by 2 °C (measured mean at 5 cm height: +0.55 °C, Schädler et al. 2019), while the amount of precipitation was altered by +10% in spring (March to May) and fall (September to November) and by -20% in summer (June to August). These manipulations follow regional climate model projections for 2070–2100 and were achieved using open, house-shaped steel constructions equipped with retractable translucent plastic tarpaulins and irrigation systems (Fig. S1, for more details see Schädler et al. 2019). In this study, the focus is on the non-fertilized extensively used meadows and pastures sown in spring 2014 with seeds of 56 native grassland species. From 2015 onward, the meadows were mown twice a year (late spring, mid-summer) with a rotary mower to a height of approx. 5 cm. The pastures were also mown in late spring 2015, while the short-term grazing started in mid-summer of the same year and was increased to three grazing events per year from 2017 onward (early spring, late spring, mid-summer). For the short-term grazing, 10–13 adult sheep and 10–20 lambs grazed each subplot for 24 h (in rotating order), summing to 10 grazing days per event. Due to insufficient biomass regrowth, mid-summer mowing and grazing were suspended in 2018, 2019, 2020, and 2022, as was early spring grazing in 2022 and mid-summer mowing in 2023 (for more details see Korell et al. 2024).

### Data collection

Directly before mowing and grazing in late spring (mostly in early June) and mid-summer (mostly in August), aboveground biomass was clipped approx. 2 cm above soil surface on four rectangles of 20 × 50 cm randomly placed in the central area of each subplot. Samples were sorted by species and detached dead plant material, dried at 70 °C for 48 h and weighed. Biomass of the four samples per subplot and harvest were averaged per species and scaled to an area of one m^2^. Community biomass (= lived biomass) was calculated as the sum across species.

In addition, the biomass removed by each mowing and grazing event was determined. In the meadows, fresh weight of mown biomass was quantified for an area of 14.4 m² (9.0 × 1.6 m) and converted to dry weight based on two dried subsamples (approx. 300 g) in each subplot. In the pastures, four grazing exclosure cages (approx. 1 m²) were placed at random positions on each subplot before grazing. Directly after grazing, four biomass samples were collected inside and outside the cages as described above. Biomass removal by grazing was estimated as the difference between ungrazed and grazed biomass.

### Biomass variability and stabilizing effects

Temporal variability of community biomass was quantified by the coefficient of variation (CV), which is defined as the standard deviation (SD) divided by the mean. Lower values indicate higher stability. The framework by Segrestin et al. (2024) was used to quantify total stabilization and partition it into three stabilizing effects: dominance, asynchrony, and averaging (for more details see Online Resource). Lower values indicate higher stabilization, while values larger than one imply destabilization.

### Background climate variability

Based on temperature data from weather stations at the field site (Gründling et al. 2022; DWD Climate Data Center 2024), potential evapotranspiration was calculated in R using the “hargreaves” function of the “SPEI” package (Hargreaves and Samani 1985; Beguería and Vicente-Serrano 2023). Atmospheric water deficit was determined as the difference between precipitation measured by the weather stations (1994–2014) or within the GCEF (2015–2023) and potential evapotranspiration. A few gaps in the precipitation data of the GCEF (3% of daily records) were filled with the mean per climate treatment or with data of the weather stations. Water deficit was aggregated over a range of time periods before each biomass harvest (1, 2, 3, 6, 9, and 12 months, May or August as the last month) to assess whether impacts on grassland biomass depend on the considered time period. The temporal variability of water deficit was quantified with the coefficient of variation (see above).

### Biomass resistance to and recovery from droughts

Standardized Precipitation-Evapotranspiration Indices (SPEI, Vicente-Serrano et al. 2010) were used to classify the time periods before each biomass harvest as normal, dry, or wet according to the long-term climate (30 years, 1994–2023). SPEI values were calculated in R with the “spei” function of the “SPEI” package (Beguería and Vicente-Serrano 2023). Following Isbell et al. (2015), time periods were classified as normal if their SPEI values were in the interquartile range of long-term climate data (-0.67 to 0.67), as moderately dry or wet if they occurred between once in four years and once per decade (-1.28 to -0.67 or 0.67 to 1.28) and as extremely dry or wet if they occurred less than once per decade (<-1.28 or >1.28).

Biomass resistance to dry periods was assessed by relating community biomass of harvests during dry periods ($\:{Biomass}_{d}$) to average normal community biomass of these harvests ($\:{Biomass}_{n}$, Bazzichetto et al. 2024):

1 $$\:Resistance=\mathrm{l}\mathrm{n}\left({Biomass}_{d}/{Biomass}_{n}\right)$$

Accordingly, resistance quantifies the deviation of community biomass from normal levels during dry periods; values are zero if no biomass has been lost and decrease with lower resistance.

Biomass recovery from dry periods was assessed by relating community biomass of harvests following dry periods ($\:{Biomass}_{d+1}$) to that during dry periods ($\:{Biomass}_{d}$, Bazzichetto et al. 2024). Because the two harvests (spring and summer) differed in biomass, biomass values were normalized by the respective average normal community biomass ($\:{Biomass}_{n+1}$, $\:{Biomass}_{n}$):

2 $$\begin{aligned}Recovery &=\mathrm{l}\mathrm{n}(\left({Biomass}_{d+1}/{Biomass}_{n+1}\right)\\&\qquad /\left({Biomass}_{d}/{Biomass}_{n}\right)) \end{aligned}$$

Recovery quantifies the extent to which community biomass has returned to normal levels after the dry period until the following harvest within a normal period; values are zero if no recovery has taken place and increase with higher recovery.

### Statistical analyses

Data were analyzed using linear mixed-effects models (“glmmTMB” package, Brooks et al. 2017) in R 4.3.3 (R Core Team 2024). First, temporal variability of community biomass and stabilizing effects were calculated for the entire time series (2015–2023) across both or single harvests, and effects of climate treatment (two levels: ambient and future), grassland management treatment (two levels: meadow and pasture), and their interaction were analyzed. To account for the experimental design, the main plot was incorporated as a random effect. Depending on the distribution of the residuals, linear or generalized linear mixed-effects models were calculated. Starting from a null model containing only random effects, the fixed effects were sequentially added. Models were fitted with the maximum likelihood method and log-likelihood ratio tests were used to test the statistical significance of the fixed effects. Post hoc comparisons were performed using the “emmeans” package (Lenth 2024).

Second, the nine-year time series was divided into overlapping three-year moving windows (shifted by one year), and temporal variability of community biomass, stabilizing effects as well as temporal variability of water deficit were calculated for each time window across both or single harvests. Effects of water deficit variability and its interactions with climate treatment and grassland management treatment on biomass variability and stabilizing effects were analyzed for each time period over which water deficit had been aggregated with the subplot nested in the main plot as random effects. Because most significant effects were found when considering the three-month period before harvest, only results for this time period are reported.

Besides, effects of the experimental treatments, year, and their interactions on community biomass resistance to and recovery from dry periods were analyzed for each harvest with the subplot nested in the main plot as random effects. Because moderate dry periods did not lead to a reduction in community biomass compared to normal periods, only results for extreme dry periods are presented.

While summer harvests consistently took place on the same dates in meadows and pastures, spring harvest dates partly differed between meadows and pastures due to organizational constraints (2017, 2020), and in addition, early spring grazing before the harvest could have influenced results for spring. To check this, model calculations for biomass variability, resistance, and recovery were repeated with adjusted community biomass data of spring harvests (Online Resource). For the adjustments, biomass uptake during early spring grazing, estimated using data from the grazing exclosure cages (see above), was added to pasture community biomass of spring harvests, and the two years with different harvest dates were excluded from the analyses.

## Results

In our grasslands, community biomass was higher in spring than in summer and varied among the nine years, with lowest spring values in 2020 and lowest summer values in 2018, 2019, 2020, and 2022 (Fig. 2a, b). In spring, mowing removed more biomass than grazing, while in summer, biomass removal was generally low (Fig. 2c, d). The springs of 2015, 2020, and 2022 and the summers of 2018, 2019, 2020, and 2022 were extremely dry compared to the last 30 years as indicated by SPEI values smaller than -1.28 (Fig. 2e, f). Except in 2020, SPEI values of extreme dry summers were distinctly lower than those of extreme dry springs suggesting stronger droughts in summer than in spring.

### Effects of climate and grassland management treatments on biomass variability and stabilizing effects

**Fig. 3:**
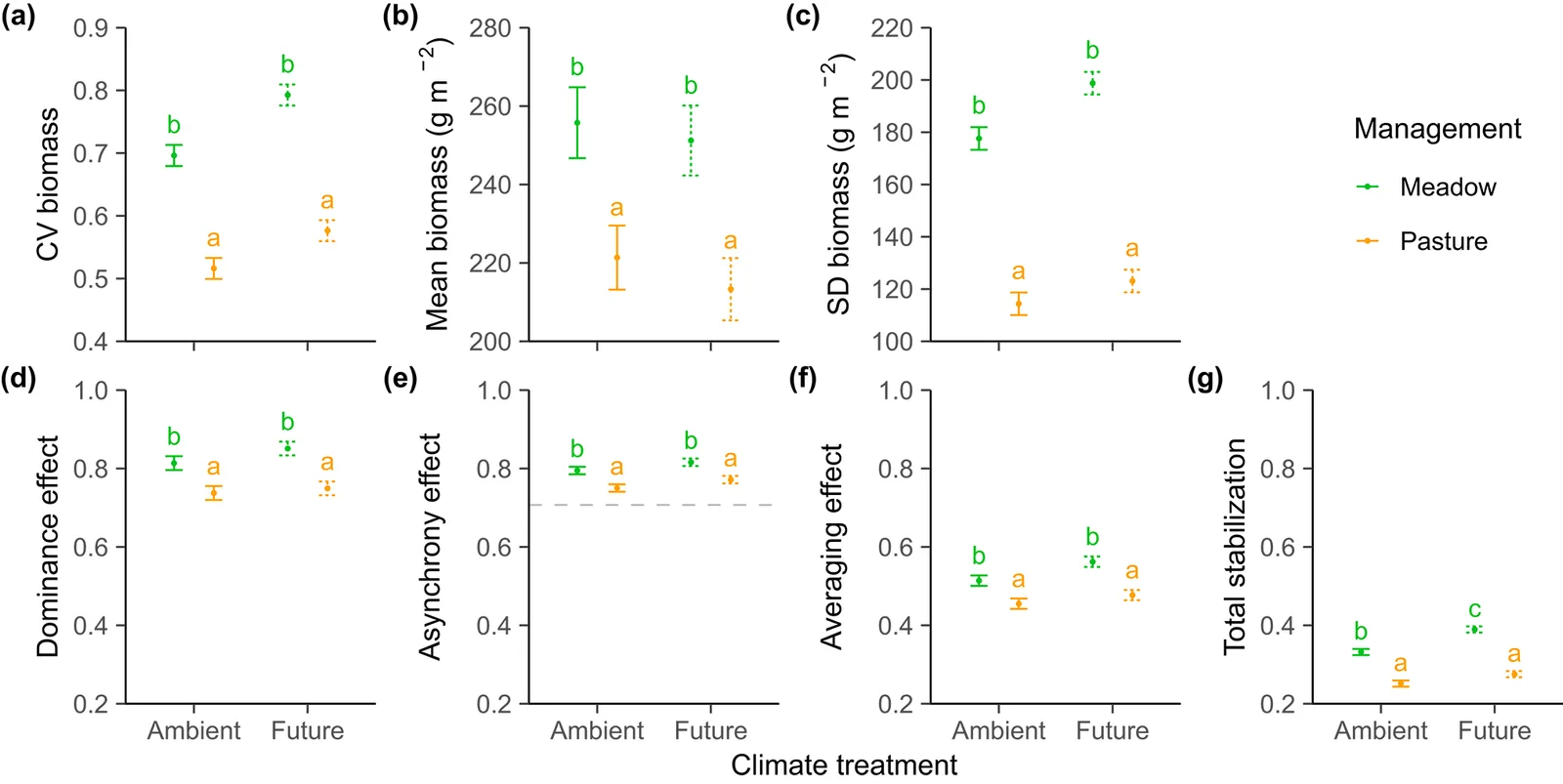
Temporal (**a**) variability (CV), (**b**) mean, (**c**) standard deviation (SD), stabilization by (**d**) dominance, (**e**) asynchrony, (**f**) averaging, and (**g**) total stabilization of community biomass per climate × grassland management treatment combination (predicted means ± SE, *n* = 5), calculated over nine years (2015–2023) across spring and summer harvests. Different lowercase letters denote significant differences (*P* < 0.05, Table 1). Lower values in (**d**-**g**) indicate higher stabilization. The dashed gray line in (**e**) represents the theoretical value of $\:\sqrt{1/2}$ corresponding to independent species fluctuation, while values above this line indicate synchronous and below asynchronous species fluctuations

Temporal variability of community biomass generally did not differ between climate treatments. However, across both harvests (spring and summer), variability as well as standard deviation of community biomass were higher in meadows than in pastures, while biomass mean was equal when adjusting for early spring grazing and different spring harvest dates (Table 1, S1, Fig. 3a-c, S2). Considering spring separately, after the adjustments, biomass variability did not differ anymore between grassland management types (Table S2, S3, Fig. S3a-c, S4). In summer, biomass variability was higher, mean was lower, and standard deviation higher in meadows than in pastures (Table S4, Fig. S5a-c).

**Table 1:** Effects of climate (CL) and grassland management (MGT) treatments as well as their two-way interaction on temporal variability (CV), mean, standard deviation (SD), stabilization by dominance, asynchrony, averaging, and total stabilization of community biomass, calculated over nine years (2015–2023) across spring and summer harvests. Results are based on a generalized linear mixed-effects model (mean) and linear mixed-effects models (CV, SD, dominance, asynchrony, averaging, total). Bold *P* values (< 0.05) indicate significance. df = degrees of freedom

		CV biomass				Mean biomass (g m^− 2^)				SD biomass (g m^− 2^)			
	df	χ²		*P*		χ²		*P*		χ²		*P*	
CL	1	2.545		0.111		0.293		0.588		0.834		0.361	
MGT	1	42.272		**< 0.001**		18.658		**< 0.001**		50.775		**< 0.001**	
CL × MGT	1	2.032		0.154		0.251		0.616		1.965		0.161	

		Dominance effect			Asynchrony effect			Averaging effect			Total stabilization		
	df	χ²	*P*		χ²		*P*	χ²	*P*		χ²		*P*
CL	1	0.833	0.361		2.209		0.137	2.609	0.106		2.821		0.093
MGT	1	16.131	**< 0.001**		18.926		**< 0.001**	17.774	**< 0.001**		40.235		**< 0.001**
CL × MGT	1	0.522	0.470		0.000		0.985	1.095	0.296		7.051		**0.008**

**Fig. 4:**
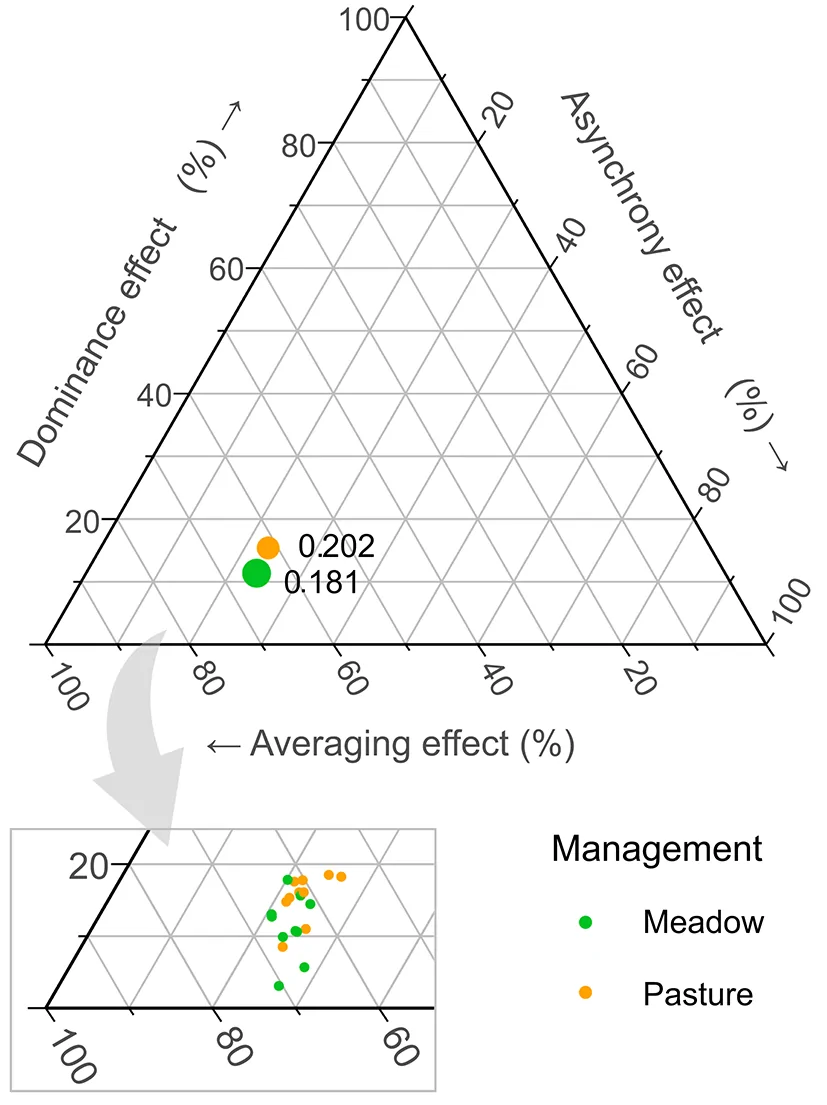
Relative contributions of dominance, asynchrony, and averaging effects to total stabilization of community biomass per grassland management treatment (means across climate treatments, *n* = 10), calculated over nine years (2015–2023) across spring harvests (summer: Fig. S6). The points are sized according to total stabilization, with the corresponding values displayed (lower values indicate higher stabilization). The magnified section shows the raw data (climate treatments not distinguished)

In spring and summer, the averaging effect generally contributed most to community biomass stabilization (63.2%, 64.0%), while the asynchrony (23.4%, 24.0%) and dominance effects (13.4%, 12.0%) were less important (Fig. 4, S6). Across both harvests, absolute values of total stabilization were higher (indicating lower stabilization) in meadows under future than ambient climate. In addition, stabilization by all three stabilizing effects and total stabilization were lower in meadows than in pastures (Table 1, Fig. 3d-g). In spring alone, total stabilization was generally lower under future compared to ambient climate. Moreover, in spring, stabilization by dominance was lower in meadows than in pastures, stabilization by asynchrony did not differ between grassland management types, and stabilization by averaging as well as total stabilization were higher in meadows than in pastures (Table S2, Fig. S3d-g). In summer, the future climate treatment had no effect, while stabilization by all stabilizing effects and total stabilization were lower in meadows than in pastures (Table S4, Fig. S5d-g).

### Effects of background climate variability on biomass variability and stabilizing effects

**Fig. 5:**
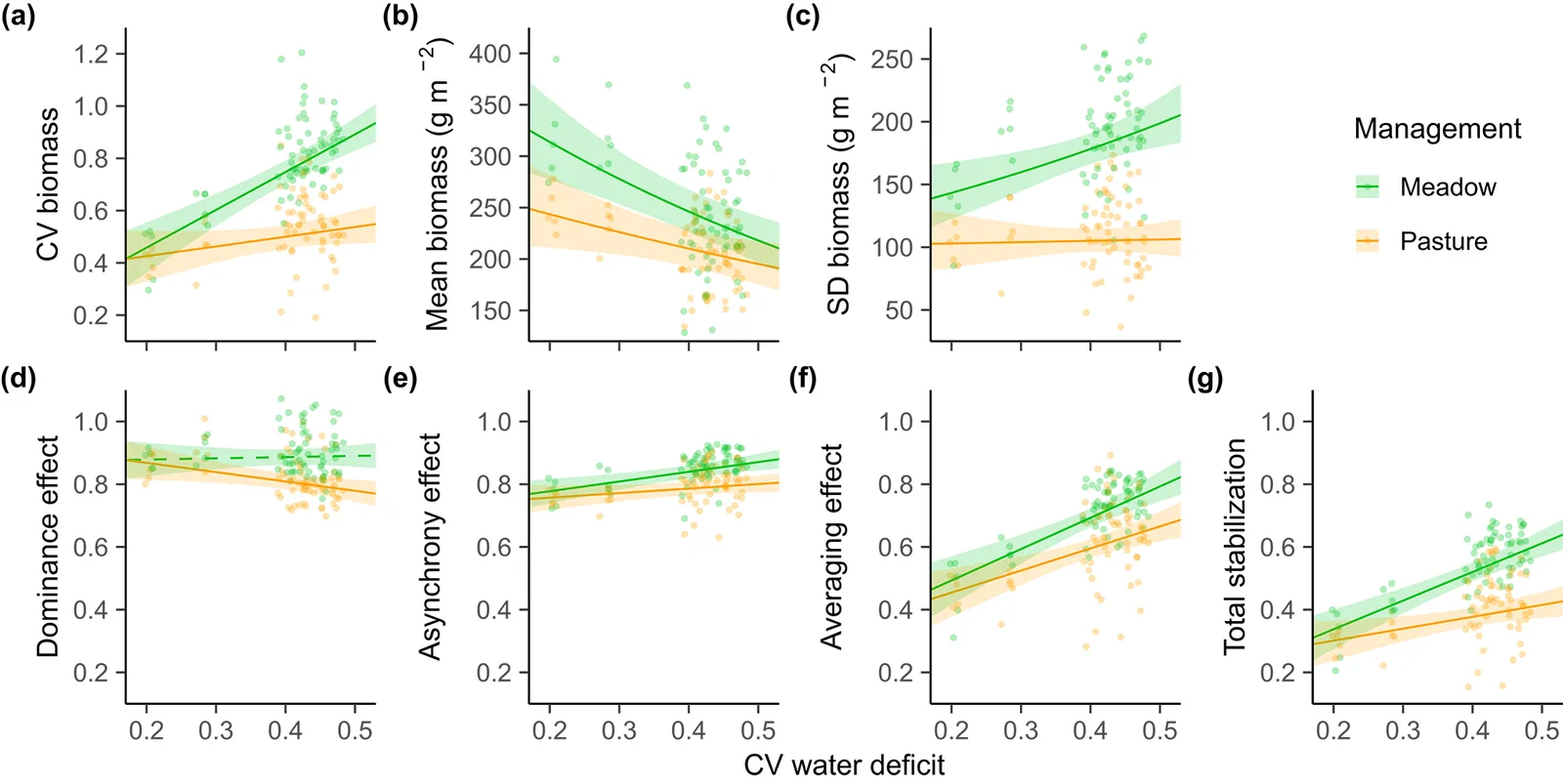
Temporal (**a**) variability (CV), (**b**) mean, (**c**) standard deviation (SD), stabilization by (**d**) dominance, (**e**) asynchrony, (**f**) averaging, and (**g**) total stabilization of community biomass dependent on temporal variability (CV) of water deficit per grassland management treatment (across climate treatments, regression lines ± 95% CI, *n* = 70), calculated for seven overlapping time windows of three years (2015–2023) across spring and summer harvests. Solid regression lines denote significant linear relationships (*P* < 0.05, Table 2). Background points show the raw data (climate treatments not distinguished). Lower values in (**d**-**g**) indicate higher stabilization, while values above one imply destabilization

Across both harvests (spring and summer), higher temporal variability of water deficit led to higher temporal variability of community biomass and the effect was stronger in meadows than in pastures. At the same time, biomass mean decreased, especially in meadows, and standard deviation increased. The latter, however, was only marginally significant after adjusting for early spring grazing and different spring harvest dates (Table 2, S5, Fig. 5a-c, S7). In spring alone, after the adjustments, variability of water deficit no longer affected biomass variability (Table S6, S7, Fig. S8a-c, S9). In summer, variability of water deficit generally enhanced biomass variability, while mean and standard deviation also increased. However, at the level of single management types, the effect on biomass variability was only significant in meadows (Table S8, Fig. S10a-c).

**Table 2:** Effects of temporal variability (CV) of water deficit (WD), climate (CL), and grassland management (MGT) treatments as well as their two- and three-way interactions on temporal variability (CV), mean, standard deviation (SD), stabilization by dominance, asynchrony, averaging, and total stabilization of community biomass, calculated for seven overlapping time windows of three years (2015–2023) across spring and summer harvests. Results are based on generalized linear mixed-effects models (mean, SD) and linear mixed-effects models (CV, dominance, asynchrony, averaging, total). Bold *P* values (< 0.05) indicate significance. df = degrees of freedom

		CV biomass				Mean biomass (g m^− 2^)				SD biomass (g m^− 2^)			
	df	χ²		*P*		χ²		*P*		χ²		*P*	
CV WD	1	36.074		**< 0.001**		24.881		**< 0.001**		5.844		**0.016**	
CL	1	1.334		0.248		0.227		0.634		1.187		0.276	
CV WD × CL	1	0.054		0.816		0.559		0.455		2.275		0.131	
MGT	1	41.991		**< 0.001**		16.929		**< 0.001**		46.246		**< 0.001**	
CV WD × MGT	1	15.227		**< 0.001**		1.871		0.171		3.678		0.055	
CL × MGT	1	0.002		0.969		0.056		0.813		0.155		0.693	
CV WD × CL × MGT	1	0.218		0.640		0.797		0.372		4.300		**0.038**	

		Dominance effect			Asynchrony effect			Averaging effect			Total stabilization		
	df	χ²	*P*		χ²		*P*	χ²	*P*		χ²		*P*
CV WD	1	6.569	**0.010**		27.274		**< 0.001**	53.817	**< 0.001**		46.528		**< 0.001**
CL	1	2.037	0.153		1.552		0.213	0.986	0.321		1.792		0.181
CV WD × CL	1	2.192	0.139		2.170		0.141	0.902	0.342		0.783		0.376
MGT	1	17.931	**< 0.001**		17.398		**< 0.001**	22.565	**< 0.001**		40.131		**< 0.001**
CV WD × MGT	1	4.944	**0.026**		2.521		0.112	1.721	0.190		8.826		**0.003**
CL × MGT	1	0.299	0.584		0.501		0.479	0.906	0.341		0.076		0.783
CV WD × CL × MGT	1	3.311	0.069		2.030		0.154	2.970	0.085		0.839		0.360

Across both harvests, higher variability of water deficit generally led to lower total stabilization, stabilization by asynchrony and averaging but higher stabilization by dominance. However, the positive effect on stabilization by dominance was only significant in pastures at the level of single management types, and total stabilization decreased more strongly in meadows than in pastures (Table 2; Fig. 5d-g). In spring or summer alone, variability of water deficit also generally decreased total stabilization, stabilization by asynchrony and averaging, and increased stabilization by dominance. However, in spring, the negative effect on total stabilization and stabilization by averaging was only significant in pastures at the level of single management types, and the positive effect on stabilization by dominance was more pronounced in meadows (Table S6, Fig. S8d-g). In summer, the negative effect on total stabilization and stabilization by asynchrony was only significant in meadows at the level of single management types, and the decrease in stabilization by averaging was more pronounced in meadows (Table S8, Fig. S10d-g).

The climate treatments generally did not influence the relationships between variability of water deficit and biomass variability or stabilizing effects.

### Effects of climate and grassland management treatments on biomass resistance to and recovery from droughts

**Fig. 6:**
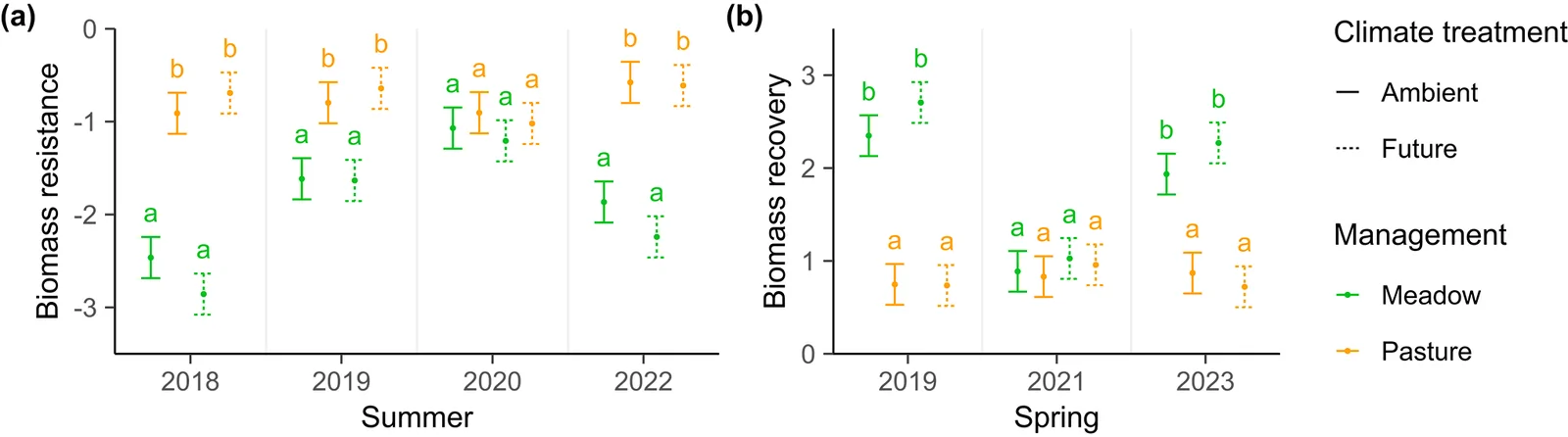
Community biomass (**a**) resistance to and (**b**) recovery from extreme dry summers per climate × grassland management treatment combination (predicted means ± SE, *n* = 5). Recovery was assessed in the following springs, provided these were classified as normal in terms of water conditions, which was not the case in 2020. Different lowercase letters denote significant differences (*P* < 0.05, Table 3)

Values of biomass resistance to extreme dry springs ranged around zero indicating that community biomass was only slightly reduced, unchanged or even higher compared to normal springs (Table S9, S10, Fig. S11a, S12a). Consequently, biomass recovery from an extreme dry spring until the following normal summer (only 2015) also showed values around zero (Table S9, Fig. S11b). In contrast, values of resistance to extreme dry summers were mostly below zero indicating a reduction in community biomass compared to normal summers. In 2018, 2019, and 2022 (but not in 2020), biomass resistance to extreme dry summers was lower in meadows than in pastures (Table 3; Fig. 6a). Biomass recovery from extreme dry summers until the following normal springs (not 2020) was higher in meadows compared to pastures in 2019 and 2023, but did not differ between management types in 2021 (Table 3, S10, Fig. 6b, S12b). Neither biomass resistance to nor biomass recovery from extreme dry summers were affected by the future climate treatment.

**Table 3:** Effects of climate (CL), grassland management (MGT), and year as well as their two- and three-way interactions on community biomass resistance to and recovery from extreme dry summers. Recovery was assessed in the following springs, provided these were classified as normal in terms of water conditions. Results are based on linear mixed-effects models. Bold *P* values (< 0.05) indicate significance. df = degrees of freedom

	Resistance to extreme dry summers			Recovery from extreme dry summers		
	df	χ²	*P*	df	χ²	*P*
CL	1	0.091	0.763	1	0.221	0.638
MGT	1	28.173	**< 0.001**	1	22.875	**< 0.001**
CL × MGT	1	1.119	0.290	1	0.673	0.412
Year	3	16.163	**0.001**	2	13.410	**0.001**
CL × Year	3	0.688	0.876	2	0.046	0.977
MGT × Year	3	35.493	**< 0.001**	2	29.986	**< 0.001**
CL × MGT × Year	3	1.400	0.705	2	0.805	0.669

## Discussion

### Effects of future climate treatment

In contrast to our first hypothesis, the future climate treatment had only little effects on stability of grassland biomass. Total stabilization was lower under future compared to ambient climate in both management types across spring harvests and only in meadows across spring and summer harvests. The greater sensitivity of meadows aligns with our second hypothesis, but otherwise meadows and pastures did not differ in their responses to the future climate treatment. In comparison to the future climate treatment, background climate variability, which included some exceptionally severe summer droughts, had more effects on stability of grassland biomass (see below). Such extreme summer droughts may become more frequent in Germany under future climate (Stanley et al. 2023). Accordingly, in line with some other recent grassland studies (Carroll et al. 2021; Korell et al. 2024), our results suggest that extreme drought events, which are also part of climate change, are stronger drivers of ecosystem dynamics than the projected changes in mean climate variables.

### Effects of background climate variability

Higher background climate variability (i.e., variability of water deficit) generally increased the temporal variability of community biomass in summer and across both harvests, which was at least partly caused by higher standard deviations contributing together with lower means to higher biomass variability (across both harvests) or offsetting higher means (summer). This is in accordance with our first hypothesis and with other studies on temperate grasslands in northern China, which found that higher natural precipitation variability increased temporal variability of community biomass (Zhang et al. 2018, 2022a, b). In addition, consistent with our hypothesis, higher variability of water deficit generally led to lower total stabilization as well as lower stabilization by asynchrony and averaging across all harvests. Obviously, the variability of water deficit was extreme enough to similarly affect species and reduce species diversity resulting in the lower stabilization by asynchrony and averaging. Accordingly, a previous study in the same experiment found lower species diversity in the dry years (Korell et al. 2024). Other time series analyses on temperate grasslands also revealed negative effects of natural precipitation variability on species asynchrony (Zhang et al. 2018, 2022a) and species richness (Zhang et al. 2022b), but did not distinguish between species asynchrony and averaging. Moreover, we found that higher variability of water deficit generally increased stabilization by dominance, however, the increase was not large enough to compensate for the lower stabilization by asynchrony and averaging. Also, previous studies on temperate and arid grasslands detected no (Hallett et al. 2014; Zhang et al. 2022a) or positive (Zhang et al. 2022b) effects of higher natural precipitation variability on dominant species stability, indicating that dominant species can be comparatively insensitive to or even benefit from climate variability.

Confirming our second hypothesis, higher background climate variability more strongly increased temporal variability of community biomass and more strongly decreased stabilization in meadows than in pastures across summers and both harvests. Biomass resistance to extreme dry summers was indeed lower in meadows than in pastures, while spring droughts generally had little impacts on aboveground biomass (resistance values around zero), which might be related to their lower drought severity (Fig. 2e-f, Matos et al. 2020; Smith et al. 2024), a well-replenished soil moisture storage from the previous winter, and higher drought resistance of plants during peak growth (Hahn et al. 2021). The lower biomass resistance to summer droughts in our meadows compared to our pastures can possibly be explained by different amounts of biomass removal in spring. While only stubbles were left after mowing (18.6%), around 62.2% of aboveground biomass remained standing after grazing in spring. The higher biomass removal in meadows than in pastures could have amplified water scarcity for plants, as more water evaporates from the soil when plant cover and vegetation height are low (Vogel et al. 2012), which could have caused the stronger biomass decline in meadows than in pastures during summer droughts. In accordance, other studies found lower drought resistance in grasslands with lower plant cover and vegetation height, regardless of whether biomass was previously removed by mowing or grazing (Vogel et al. 2012; Deléglise et al. 2015). In our meadows, the lower biomass resistance to summer droughts was followed by a higher biomass recovery compared to our pastures. There is increasing evidence that a lower biomass resistance is often associated with a higher biomass recovery (e.g., Matos et al. 2020). Lower biomass resistance during drought can lead to higher microbial activity and a stronger pulse in nutrient availability after rewetting, which can promote plant growth (Mackie et al. 2019; van Sundert et al. 2020; Hahn et al. 2021).

### Importance of stabilizing effects

The diversity-associated averaging effect generally contributed most to total stabilization of community biomass in our high-diversity grasslands, while the dominance and asynchrony effects were less important. This is in accordance with our third hypothesis and with results from a removal experiment in a diverse wet meadow applying the same partitioning framework (Lisner et al. 2024; Segrestin et al. 2024) and suggests that statistical averaging of species fluctuations may be more important for temporal stability of grassland biomass than the degree of asynchrony.

Inconsistent with our third hypothesis, across both seasons and summers alone, stabilization by all stabilizing effects as well as total stabilization were lower in meadows than in pastures resulting in higher temporal variability of community biomass due to significantly higher standard deviations (across both harvests) and lower means (summer). This can be explained by the fact that lower biomass resistance to summer droughts in meadows than pastures necessarily results in higher biomass variability. In contrast, in line with our third hypothesis, stabilization by dominance was lower, while stabilization by averaging was higher in meadows than in pastures in spring at peak biomass production resulting in a slightly higher total stabilization in meadows compared to pastures (Fig. 4, S3). However, biomass variability was no longer lower in meadows than in pastures after adjusting for early spring grazing and different spring harvest dates. The lower stabilization by dominance and the higher stabilization by averaging in meadows than in pastures might be related to the low-intensity grazing, which removed low amounts of biomass and allowed the sheep to forage selectively, gradually reducing species diversity and increasing the dominance of tall-growing and less palatable species (Koerner et al. 2018; Eskelinen et al. 2022; Han et al. 2023). In addition, the twice-yearly mowing could have decreased dominance and promoted species diversity by reducing light competition (Zhao et al. 2024). Consistently, a previous study in the same experiment found less decreases in evenness and higher species richness in meadows than in pastures (Korell et al. 2024). Continued development toward greater dominance and reduced species diversity in our low-intensity grazed pastures could further decrease stabilization by averaging with consequences for biomass stability, probably counteracting the buffering effect of low biomass removal in the long term. Accordingly, studies on grasslands in northern China have related lower biomass stability on grazed sites to increased species dominance and reduced stabilization by species diversity (Ren et al. 2018; Han et al. 2023; Zhao et al. 2024). In our study and in contrast to our hypothesis, stabilization by asynchrony did not differ between grassland management types in spring. Similarly, Zhang et al. (2016) also found no difference in species covariance between meadows and pastures. However, in other studies, species asynchrony was indeed higher on mown or mown-and-grazed sites than on exclusively grazed sites (Ren et al. 2018; Zhao et al. 2024), but they did not distinguish between species asynchrony and averaging, so that the higher species asynchrony could have been due to higher species averaging.

## Conclusions

Our findings suggest that high-diversity grasslands can buffer moderate changes in seasonal climate variability, but that high inter-annual climate variability including extreme droughts can destabilize their biomass due to reduced asynchrony and averaging of species fluctuations. Lower biomass removal through management can increase biomass resistance to extreme drought events. However, insufficient management intensity with biomass accumulation and increased light competition can also promote species loss and thus weaken stabilization by the diversity-associated averaging effect, probably counteracting the buffering effect of low biomass removal in the long term. We conclude that both a protective plant cover during drought and species diversity are important to ensure stable biomass production in managed anthropogenic grasslands under changing climatic conditions.

## Supplementary Information

Below is the link to the electronic supplementary material.

Supplementary Material 1

## Data Availability

The datasets analyzed during this study and the code are available from the corresponding author on reasonable request.
