# Supplementary material for: Climate variability reduces biomass stability in species-rich grasslands, depending on management
Source: Oecologia. 2026 Jul 16;208(8):93. doi: 10.1007/s00442-026-05925-2 (PMC13375811; doi:10.1007/s00442-026-05925-2)
Supplement: Supplementary file 1 — Supplementary Material 1 [file 442_2026_5925_MOESM1_ESM.pdf]

Supplemental material

Title:

Climate variability reduces biomass stability in species-rich grasslands, depending on management

Authors:

Yva Herion, Martin Andrzejak, Harald Auge, Walter Durka, Sylvia Haider, Johannes Höfner, Lotte Korell, Anna-Maria Madaj, Stefan Michalski, Erik Welk, Anke Hildebrandt, W. Stanley Harpole, Christiane Roscher

Corresponding author:

Yva Herion, yva.herion@ufz.de

Journal:

Oecologia

Biomass variability and stabilizing effects

In more detail, the framework by Segrestin et al. (2024) partitions temporal variability of community biomass ($CV_{com} = \sigma_{com}/\mu_{com}$, where μ_{com} is the temporal mean of community biomass and σ_{com} its temporal standard deviation) into the variability of an average species of the community (CV_e) and three stabilizing effects: dominance effect (Δ), asynchrony effect (ψ), and averaging effect (ω):

$$CV_{com} = CV_e \Delta \psi \omega$$

The stability of a theoretical average species (CV_e), whose abundance (μ_e) is equal to the average abundance of the species in the community, is estimated using Taylor's power law (Taylor 1961, $\sigma_i^2 = a\mu_i^b$), while a and b are derived from the biomass means and variances of all species in the community ($CV_e = a^{1/2}\mu_e^b$). Communities are usually more stable than the average species and total stabilization is defined as $\tau = CV_{com}/CV_e$, which can be further decomposed into the three stabilizing effects ($\tau = \Delta\psi\omega$). The dominance effect (Δ) corresponds to the stabilization by stable dominant species and is calculated as $\Delta = \widetilde{CV}/CV_e$, where $\widetilde{CV}$ is the mean of the CV_i of species i weighted by its relative abundance (p_i) in the community ($\widetilde{CV} = \sum_{i=1}^{n} p_i\, CV_i$). If b of Taylor's power law is lower than two, the stability of species increases with their abundance and a greater dominance of species has a stabilizing effect ($\Delta < 1$), otherwise ($b > 2$, $\Delta > 1$) a destabilizing effect of dominant species can be expected. The stabilization due to compensations among species is determined by the square root of the asynchrony index ($\sqrt{\varphi} = \sigma_{com}/\sum_{i=1}^{n}\sigma_i$) from Loreau and Mazancourt (2008) and is further partitioned into stabilization by asynchrony (ψ) and averaging (ω) effect ($\sqrt{\varphi} = \psi\omega$). For the asynchrony effect (ψ), $\sqrt{\varphi}$ is rescaled under the assumption of a hypothetical community with a diversity of two species and to obtain a constant value ($\sqrt{1/2} \approx 0.71$) for communities with independent species fluctuations irrespective of species diversity. $\psi > \sqrt{1/2}$ indicates prevalent synchronous, while $\psi < \sqrt{1/2}$ refers to mostly asynchronous species fluctuations. As most communities consist of more than two species, the stabilization by compensations among species is often higher than the stabilization by the “pure” asynchrony effect, and $\omega = \sqrt{\varphi}/\psi$ captures the stabilization related to the averaging effect.

Loreau M, Mazancourt C de (2008) Species synchrony and its drivers: neutral and nonneutral community dynamics in fluctuating environments. Am Nat 172:E48-66 https://doi.org/10.1086/589746.

Segrestin J, Götzenberger L, Valencia E, de Bello F, Lepš J (2024) A unified framework for partitioning the drivers of stability of ecological communities. Global Ecol Biogeogr 33:e13828 https://doi.org/10.1111/geb.13828

Taylor LR (1961) Aggregation, variance and the mean. Nature 189:732–735 https://doi.org/10.1038/189732a0.

Table S1 Effects of climate (CL) and grassland management (MGT) treatments as well as their two-way interaction on temporal variability (CV), mean, and standard deviation (SD) of community biomass, calculated over seven years (2015 – 2023, 2017 and 2020 excluded due to different spring harvest dates in the management types) across spring (biomass uptake during early spring grazing added) and summer harvests. Results are based on generalized linear mixed-effects models (CV, mean) and linear mixed-effects models (SD). Bold *P* values (< 0.05) indicate significance. df = degrees of freedom

		CV biomass		Mean biomass (g m^{-2})		SD biomass (g m^{-2})	
	df	χ^2	*P*	χ^2	*P*	χ^2	*P*
CL	1	3.020	0.082	0.108	0.742	1.740	0.187
MGT	1	34.606	**<0.001**	2.125	0.145	13.477	**<0.001**
CL × MGT	1	0.051	0.821	0.299	0.585	0.155	0.694

Table S2 Effects of climate (CL) and grassland management (MGT) treatments as well as their two-way interaction on temporal variability (CV), mean, standard deviation (SD), stabilization by dominance, asynchrony, averaging, and total stabilization of community biomass, calculated over nine years (2015 – 2023) across spring harvests. Results are based on generalized linear mixed-effects models (CV, mean, asynchrony, averaging, total) and linear mixed-effects models (SD, dominance). Bold *P* values (< 0.05) indicate significance. df = degrees of freedom

		CV biomass		Mean biomass (g m^{-2})		SD biomass (g m^{-2})	
	df	χ^2	*P*	χ^2	*P*	χ^2	*P*
CL	1	2.278	0.131	0.047	0.829	3.463	0.063
MGT	1	9.194	**0.002**	33.835	**<0.001**	1.191	0.275
CL × MGT	1	1.160	0.281	0.609	0.435	1.817	0.178

		Dominance effect		Asynchrony effect		Averaging effect		Total stabilization	
	df	χ^2	*P*	χ^2	*P*	χ^2	*P*	χ^2	*P*
CL	1	0.609	0.435	1.906	0.167	2.470	0.116	4.203	**0.040**
MGT	1	4.373	**0.037**	2.929	0.087	6.220	**0.013**	4.368	**0.037**
CL × MGT	1	0.082	0.775	0.531	0.466	1.724	0.189	2.268	0.132

Table S3 Effects of climate (CL) and grassland management (MGT) treatments as well as their two-way interaction on temporal variability (CV), mean, and standard deviation (SD) of community biomass, calculated over seven years (2015 – 2023, 2017 and 2020 excluded due to different spring harvest dates in the management types) across spring harvests (biomass uptake during early spring grazing added). Results are based on generalized linear mixed-effects models. Bold *P* values (< 0.05) indicate significance. df = degrees of freedom

		CV biomass		Mean biomass (g m^{-2})		SD biomass (g m^{-2})	
	df	χ^2	*P*	χ^2	*P*	χ^2	*P*
CL	1	0.010	0.921	0.416	0.519	0.625	0.429
MGT	1	0.700	0.403	2.439	0.118	0.003	0.954
CL × MGT	1	0.196	0.658	0.246	0.620	0.865	0.352

Table S4 Effects of climate (CL) and grassland management (MGT) treatments as well as their two-way interaction on temporal variability (CV), mean, standard deviation (SD), stabilization by dominance, asynchrony, averaging, and total stabilization of community biomass, calculated over nine years (2015 – 2023) across summer harvests. Results are based on generalized linear mixed-effects models (mean, SD, dominance, asynchrony) and linear mixed-effects models (CV, averaging, total). Bold *P* values (< 0.05) indicate significance. df = degrees of freedom

		CV biomass		Mean biomass (g m^{-2})		SD biomass (g m^{-2})	
	df	χ^2	*P*	χ^2	*P*	χ^2	*P*
CL	1	0.211	0.646	4.745	**0.029**	0.397	0.529
MGT	1	37.395	**<0.001**	26.043	**<0.001**	13.643	**<0.001**
CL × MGT	1	0.024	0.878	4.296	**0.038**	1.962	0.161

		Dominance effect		Asynchrony effect		Averaging effect		Total stabilization	
	df	χ^2	*P*	χ^2	*P*	χ^2	*P*	χ^2	*P*
CL	1	0.102	0.749	0.193	0.661	0.914	0.339	0.589	0.443
MGT	1	23.654	**<0.001**	17.647	**<0.001**	23.396	**<0.001**	34.105	**<0.001**
CL × MGT	1	2.264	0.132	1.182	0.277	1.096	0.295	0.001	0.973

Table S5 Effects of temporal variability (CV) of water deficit (WD), climate (CL), and grassland management (MGT) treatments as well as their two- and three-way interactions on temporal variability (CV), mean, and standard deviation (SD) of community biomass, calculated for five overlapping time windows of three years (2015 – 2023, 2017 and 2020 excluded due to different spring harvest dates in the management types) across spring (biomass uptake during early spring grazing added) and summer harvests. Results are based on generalized linear mixed-effects models. Bold *P* values (< 0.05) indicate significance. df = degrees of freedom

		CV biomass		Mean biomass (g m^{-2})		SD biomass (g m^{-2})	
	df	χ^2	*P*	χ^2	*P*	χ^2	*P*
CV WD	1	46.519	**<0.001**	12.118	**<0.001**	3.450	0.063
CL	1	1.345	0.246	0.145	0.704	1.945	0.163
CV WD × CL	1	0.038	0.846	0.673	0.412	0.601	0.438
MGT	1	29.294	**<0.001**	2.848	0.092	12.099	**0.001**
CV WD × MGT	1	11.022	**0.001**	11.608	**0.001**	0.008	0.930
CL × MGT	1	0.448	0.503	0.742	0.389	0.041	0.841
CV WD × CL × MGT	1	0.276	0.599	0.816	0.366	0.514	0.474

Table S6 Effects of temporal variability (CV) of water deficit (WD), climate (CL), and grassland management (MGT) treatments as well as their two- and three-way interactions on temporal variability (CV), mean, standard deviation (SD), stabilization by dominance, asynchrony, averaging, and total stabilization of community biomass, calculated for seven overlapping time windows of three years (2015 – 2023) across spring harvests. Results are based on generalized linear mixed-effects models (mean, SD) and linear mixed-effects models (CV, dominance, asynchrony, averaging, total). Bold *P* values (< 0.05) indicate significance. df = degrees of freedom

		CV biomass		Mean biomass (g m^{-2})		SD biomass (g m^{-2})	
	df	χ^2	*P*	χ^2	*P*	χ^2	*P*
CV WD	1	0.974	0.324	6.439	**0.011**	0.209	0.648
CL	1	3.426	0.064	0.499	0.480	5.273	**0.022**
CV WD × CL	1	0.000	0.993	0.471	0.492	0.000	0.987
MGT	1	3.951	**0.047**	32.181	**<0.001**	0.838	0.360
CV WD × MGT	1	9.431	**0.002**	17.341	**<0.001**	14.353	**<0.001**
CL × MGT	1	0.095	0.758	0.242	0.622	2.630	0.105
CV WD × CL × MGT	1	0.069	0.792	0.642	0.423	0.004	0.949

		Dominance effect		Asynchrony effect		Averaging effect		Total stabilization	
	df	χ^2	*P*	χ^2	*P*	χ^2	*P*	χ^2	*P*
CV WD	1	29.082	**<0.001**	14.391	**<0.001**	13.175	**<0.001**	5.566	**0.018**
CL	1	5.297	**0.021**	1.656	0.198	2.329	0.127	4.110	**0.043**
CV WD × CL	1	0.835	0.361	0.019	0.890	0.616	0.433	0.044	0.834
MGT	1	1.950	0.163	3.167	0.075	4.632	**0.031**	3.549	0.060
CV WD × MGT	1	5.683	**0.017**	0.044	0.834	14.820	**<0.001**	12.477	**<0.001**
CL × MGT	1	1.419	0.234	0.028	0.867	0.064	0.801	0.082	0.774
CV WD × CL × MGT	1	0.816	0.366	0.058	0.811	0.440	0.507	0.001	0.982

Table S7 Effects of temporal variability (CV) of water deficit (WD), climate (CL), and grassland management (MGT) treatments as well as their two- and three-way interactions on temporal variability (CV), mean, and standard deviation (SD) of community biomass, calculated for five overlapping time windows of three years (2015 – 2023, 2017 and 2020 excluded due to different spring harvest dates in the management types) across spring harvests (biomass uptake during early spring grazing added). Results are based on generalized linear mixed-effects models (mean, SD) and linear mixed-effects models (CV). Bold *P* values (< 0.05) indicate significance. df = degrees of freedom

		CV biomass		Mean biomass (g m^{-2})		SD biomass (g m^{-2})	
	df	χ^2	*P*	χ^2	*P*	χ^2	*P*
CV WD	1	1.589	0.208	0.173	0.678	0.356	0.551
CL	1	0.155	0.694	1.065	0.302	1.178	0.278
CV WD × CL	1	1.133	0.287	0.069	0.793	0.671	0.413
MGT	1	0.128	0.720	2.564	0.109	0.097	0.755
CV WD × MGT	1	0.005	0.945	13.835	**<0.001**	0.002	0.965
CL × MGT	1	0.274	0.601	0.097	0.756	0.087	0.769
CV WD × CL × MGT	1	0.004	0.953	0.041	0.839	0.011	0.915

Table S8 Effects of temporal variability (CV) of water deficit (WD), climate (CL), and grassland management (MGT) treatments as well as their two- and three-way interactions on temporal variability (CV), mean, standard deviation (SD), stabilization by dominance, asynchrony, averaging, and total stabilization of community biomass, calculated for seven overlapping time windows of three years (2015 – 2023) across summer harvests. Results are based on generalized linear mixed-effects models. Bold *P* values (< 0.05) indicate significance. df = degrees of freedom

		CV biomass		Mean biomass (g m^{-2})		SD biomass (g m^{-2})	
	df	χ^2	*P*	χ^2	*P*	χ^2	*P*
CV WD	1	33.515	**<0.001**	7.037	**0.008**	22.472	**<0.001**
CL	1	0.974	0.324	0.666	0.414	0.361	0.548
CV WD × CL	1	0.179	0.672	0.021	0.885	1.058	0.304
MGT	1	27.394	**<0.001**	34.795	**<0.001**	0.064	0.800
CV WD × MGT	1	9.941	**0.002**	2.618	0.106	2.657	0.103
CL × MGT	1	1.193	0.275	0.302	0.583	0.050	0.823
CV WD × CL × MGT	1	1.214	0.270	0.224	0.636	0.237	0.627

		Dominance effect		Asynchrony effect		Averaging effect		Total stabilization	
	df	χ^2	*P*	χ^2	*P*	χ^2	*P*	χ^2	*P*
CV WD	1	6.730	**0.009**	34.504	**<0.001**	48.784	**<0.001**	45.110	**<0.001**
CL	1	0.003	0.955	1.038	0.308	2.286	0.131	1.085	0.298
CV WD × CL	1	2.633	0.105	0.092	0.762	0.025	0.874	0.219	0.640
MGT	1	15.441	**<0.001**	21.079	**<0.001**	19.633	**<0.001**	26.018	**<0.001**
CV WD × MGT	1	1.699	0.192	10.357	**0.001**	10.060	**0.002**	12.125	**<0.001**
CL × MGT	1	1.943	0.163	0.503	0.478	0.000	0.993	0.242	0.623
CV WD × CL × MGT	1	0.033	0.857	1.950	0.163	0.510	0.475	0.788	0.375

Table S9 Effects of climate (CL), grassland management (MGT), and year as well as their two- and three-way interactions on community biomass resistance to and recovery from extreme dry springs. Recovery was assessed in the following summers, provided these were classified as normal in terms of water conditions (only 2015). Results are based on linear mixed-effects models. Bold *P* values (< 0.05) indicate significance. df = degrees of freedom

	Resistance to extreme dry springs			Recovery from extreme dry springs		
	df	χ^2	*P*	df	χ^2	*P*
CL	1	2.410	0.121	1	1.177	0.278
MGT	1	16.412	**<0.001**	1	23.224	**<0.001**
CL × MGT	1	0.002	0.962	1	3.293	0.070
Year	2	30.534	**<0.001**			
CL × Year	2	5.509	0.064			
MGT × Year	2	12.328	**0.002**			
CL × MGT × Year	2	0.203	0.903			

Table S10 Effects of climate (CL), grassland management (MGT), and year as well as their two- and three-way interactions on community biomass resistance to extreme dry springs (2020 excluded due to different spring harvest dates in the management types) and recovery from extreme dry summers. Recovery was assessed in the following springs, provided these were classified as normal in terms of water conditions. Community biomass in springs was adjusted by adding biomass uptake during early spring grazing. Results are based on linear mixed-effects models. Bold *P* values (< 0.05) indicate significance. df = degrees of freedom

	Resistance to extreme dry springs			Recovery from extreme dry summers		
	df	χ^2	*P*	df	χ^2	*P*
CL	1	2.057	0.151	1	0.207	0.649
MGT	1	0.735	0.391	1	25.273	**<0.001**
CL × MGT	1	0.061	0.804	1	0.823	0.364
Year	1	8.624	**0.003**	2	15.063	**0.001**
CL × Year	1	5.228	**0.022**	2	0.004	0.998
MGT × Year	1	4.901	**0.027**	2	29.309	**<0.001**
CL × MGT × Year	1	0.211	0.646	2	0.916	0.633

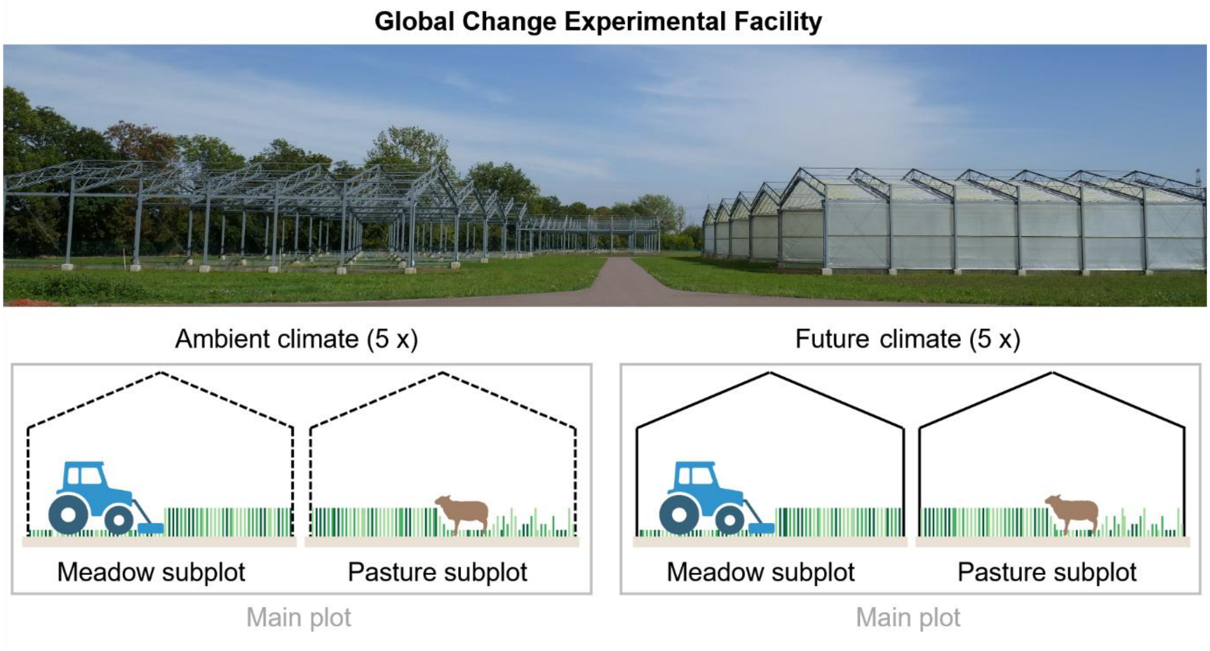

Fig. S1 Schematic overview of the experimental design. The Global Change Experimental Facility (Bad Lauchstädt, Germany) follows a randomized split-plot design with climate as the main-plot factor (ambient and future climate treatment, each replicated in five main plots) and land use as the subplot factor (five subplots of 16 × 24 m per main plot). The climate manipulation is achieved by moveable translucent plastic tarpaulins (closing roofs and sides of the house-shaped open steel constructions) and irrigation systems. This study focuses on the species-rich non-fertilized extensively used meadows and pastures, either managed by mowing (two times per year) or by sheep grazing (three times per years). This figure was partially created in BioRender: Herion, Y. (2026), https://BioRender.com/n2kt6nh

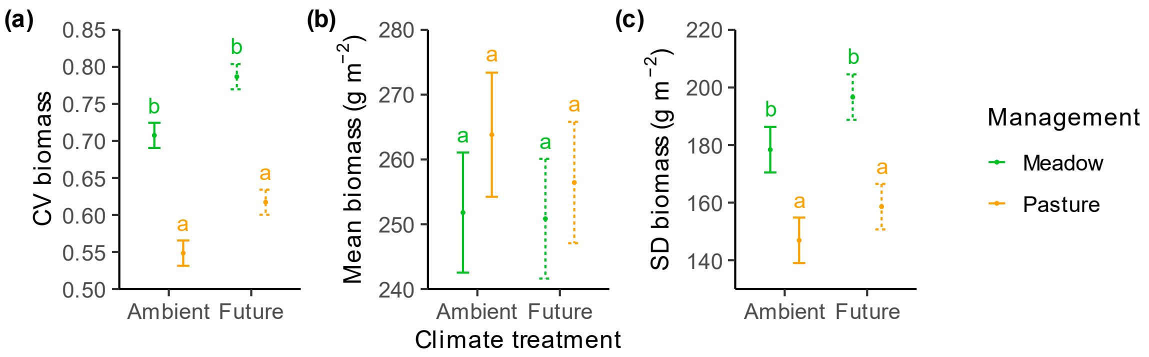

Fig. S2 Temporal (a) variability (CV), (b) mean, and (c) standard deviation (SD) of community biomass per climate × grassland management treatment combination (predicted means ± SE, n = 5), calculated over seven years (2015 – 2023, 2017 and 2020 excluded due to different spring harvest dates in the management types) across spring (biomass uptake during early spring grazing added) and summer harvests. Different lowercase letters denote significant differences (*P* < 0.05, Table S1)

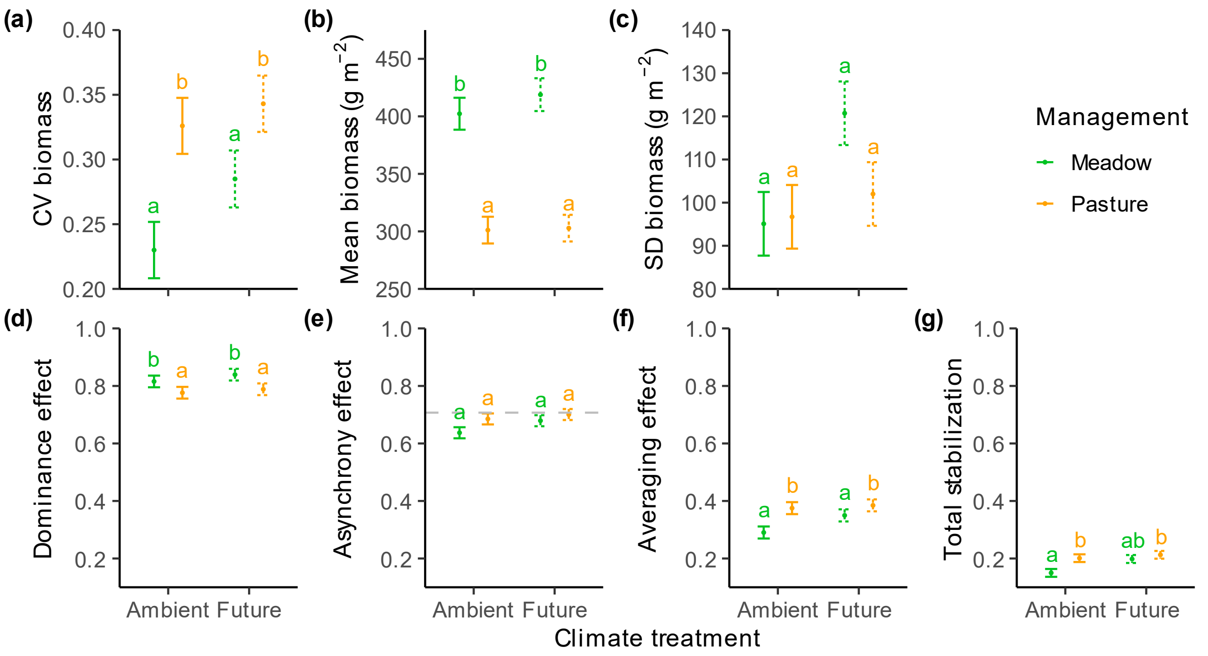

Fig. S3 Temporal (a) variability (CV), (b) mean, (c) standard deviation (SD), stabilization by (d) dominance, (e) asynchrony, (f) averaging, and (g) total stabilization of community biomass per climate × grassland management treatment combination (predicted means ± SE, n = 5), calculated over nine years (2015 – 2023) across spring harvests. Different lowercase letters denote significant differences ($P < 0.05$, Table S2). Lower values in (d-g) indicate higher stabilization. The dashed gray line in (e) represents the theoretical value of $\sqrt{1/2}$ corresponding to independent species fluctuation, while values above this line indicate synchronous and below asynchronous species fluctuations

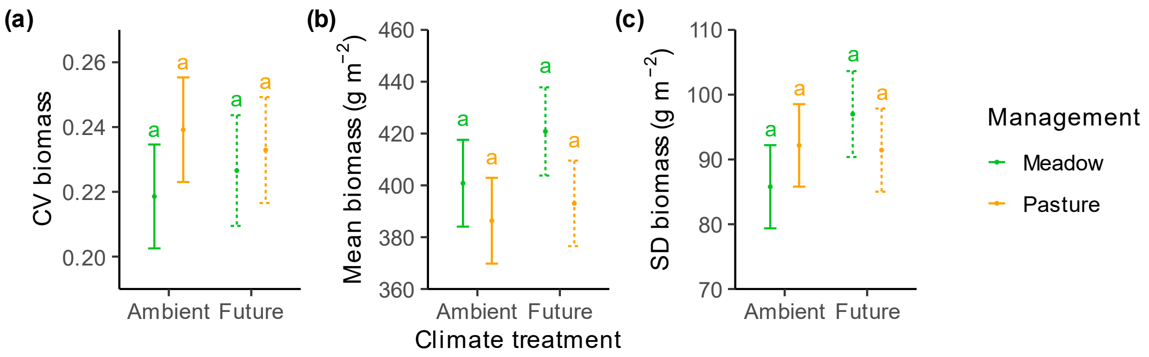

Fig. S4 Temporal (a) variability (CV), (b) mean, and (c) standard deviation (SD) of community biomass per climate × grassland management treatment combination (predicted means ± SE, n = 5), calculated over seven years (2015 – 2023, 2017 and 2020 excluded due to different spring harvest dates in the management types) across spring harvests (biomass uptake during early spring grazing added). Different lowercase letters denote significant differences ($P < 0.05$, Table S3)

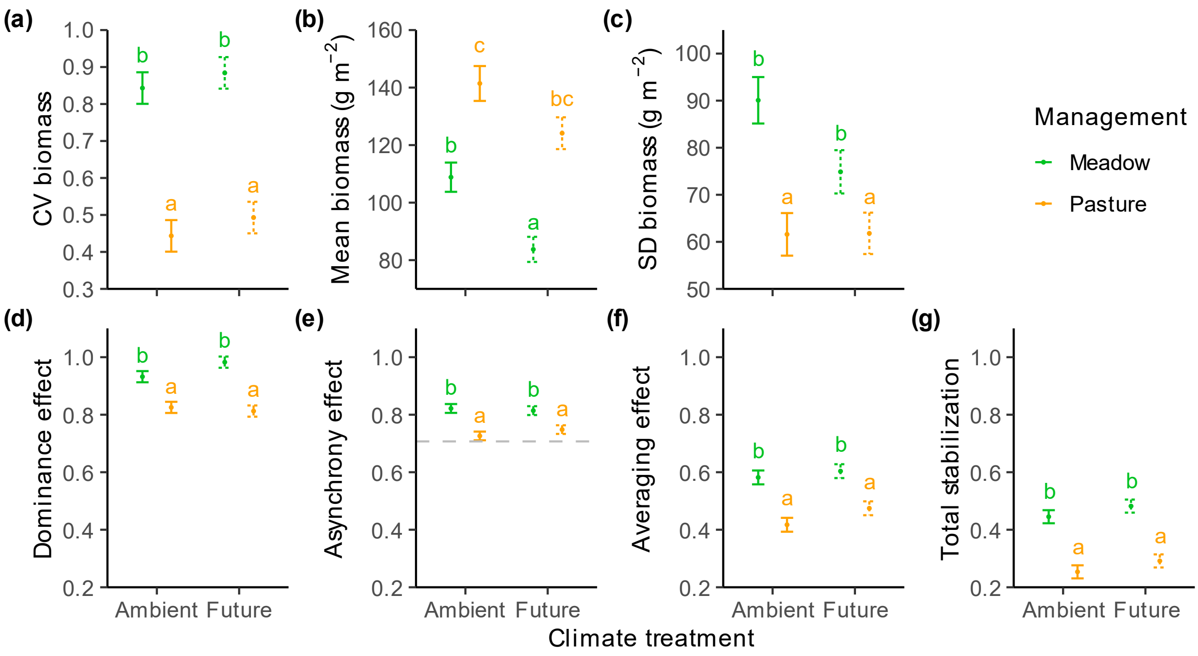

Fig. S5 Temporal (a) variability (CV), (b) mean, (c) standard deviation (SD), stabilization by (d) dominance, (e) asynchrony, (f) averaging, and (g) total stabilization of community biomass per climate × grassland management treatment combination (predicted means ± SE, n = 5), calculated over nine years (2015 – 2023) across summer harvests. Different lowercase letters denote significant differences ($P < 0.05$, Table S4). Lower values in (d-g) indicate higher stabilization, while values above one imply destabilization. The dashed gray line in (e) represents the theoretical value of $\sqrt{1/2}$ corresponding to independent species fluctuation, while values above this line indicate synchronous and below asynchronous species fluctuations

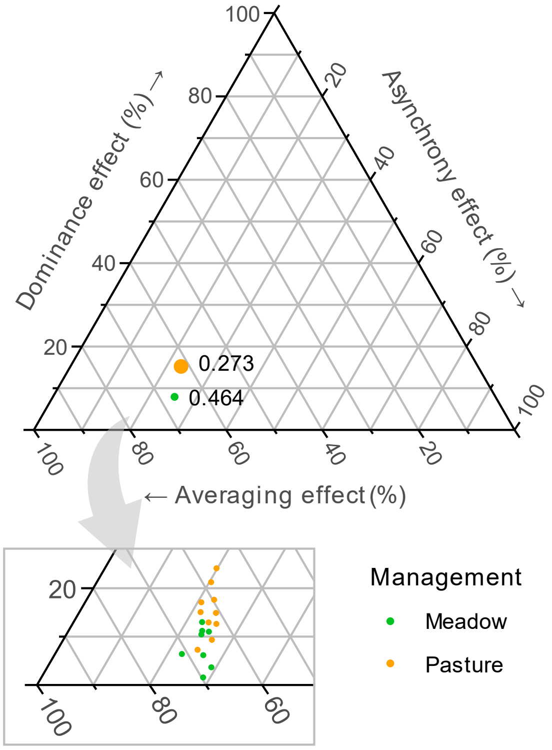

Fig. S6 Relative contributions of dominance, asynchrony, and averaging effects to total stabilization of community biomass per grassland management treatment (across climate treatments, means, n = 10), calculated over nine years (2015 - 2023) across summer harvests. The points are sized according to total stabilization, with the corresponding values displayed (lower values indicate higher stabilization). The magnified section shows the raw data (climate treatments not distinguished). The relative contributions of stabilizing effects could not be determined for two meadow subplots due to destabilization by dominance effect

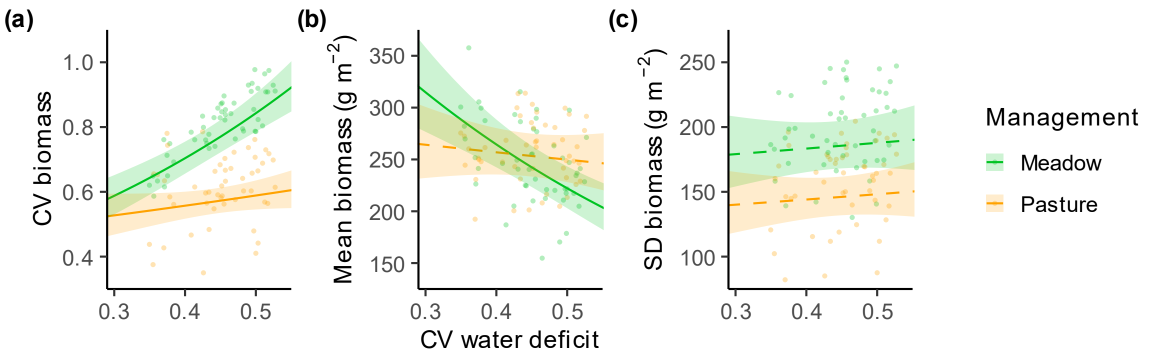

Fig. S7 Temporal (a) variability (CV), (b) mean, and (c) standard deviation (SD) of community biomass dependent on temporal variability (CV) of water deficit per grassland management treatment (across climate treatments, regression lines ± 95% CI, n = 50), calculated for five overlapping time windows of three years (2015 – 2023, 2017 and 2020 excluded due to different spring harvest dates in the management types) across spring (biomass uptake during early spring grazing added) and summer harvests. Solid regression lines denote significant linear relationships ($P < 0.05$, Table S5). Background points show the raw data (climate treatments not distinguished)

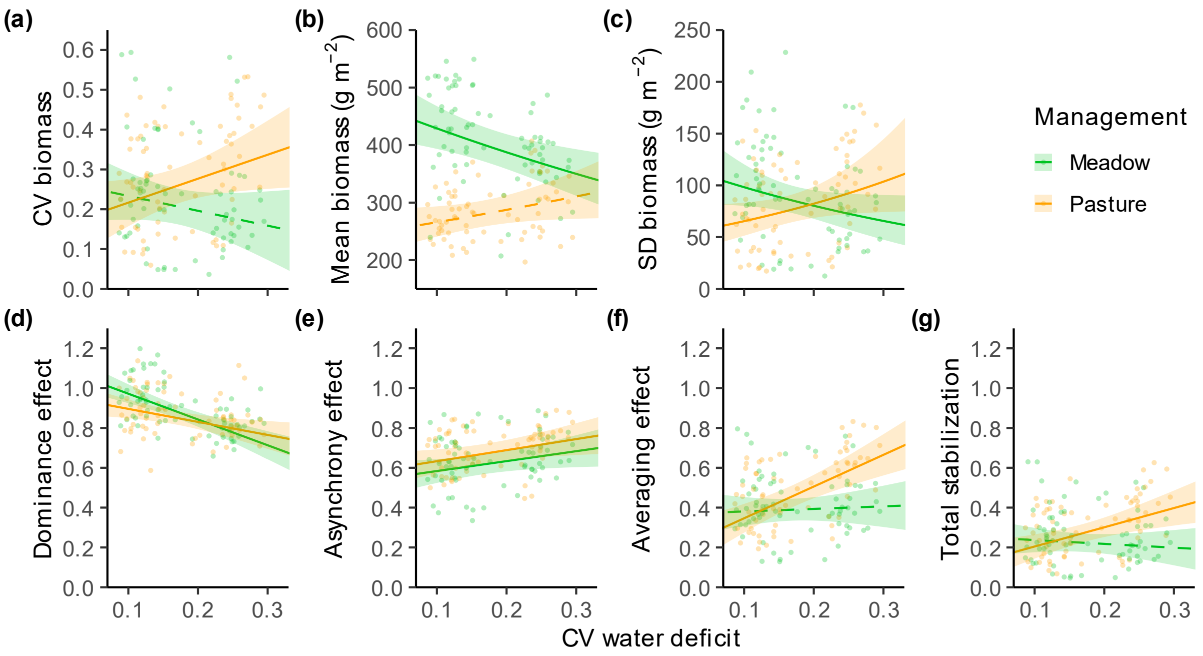

Fig. S8 Temporal (a) variability (CV), (b) mean, (c) standard deviation (SD), stabilization by (d) dominance, (e) asynchrony, (f) averaging, and (g) total stabilization of community biomass dependent on temporal variability (CV) of water deficit per grassland management treatment (across climate treatments, regression lines ± 95% CI, n = 70), calculated for seven overlapping time windows of three years (2015 – 2023) across spring harvests. Solid regression lines denote significant linear relationships (P < 0.05, Table S6). Background points show the raw data (climate treatments not distinguished). Lower values in (d-g) indicate higher stabilization, while values above one imply destabilization

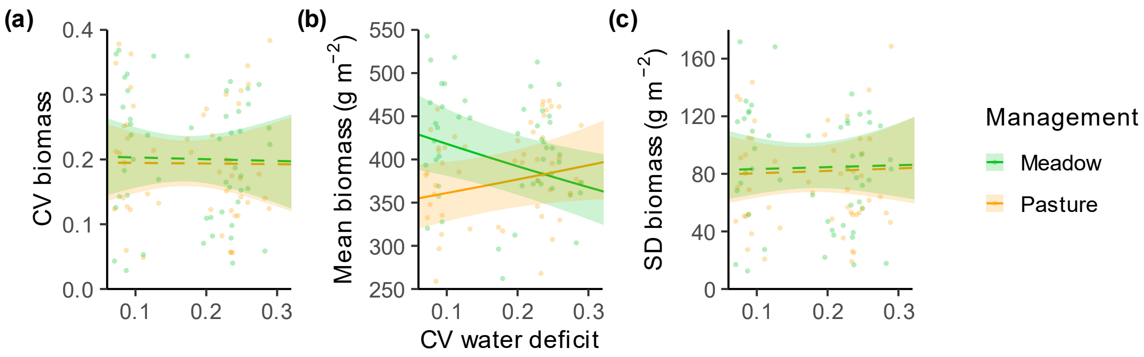

Fig. S9 Temporal (a) variability (CV), (b) mean, and (c) standard deviation (SD) of community biomass dependent on temporal variability (CV) of water deficit per grassland management treatment (across climate treatments, regression lines ± 95% CI, n = 50), calculated for five overlapping time windows of three years (2015 – 2023, 2017 and 2020 excluded due to different spring harvest dates in the management types) across spring harvests (biomass uptake during early spring grazing added). Solid regression lines denote significant linear relationships (P < 0.05, Table S7). Background points show the raw data (climate treatments not distinguished)

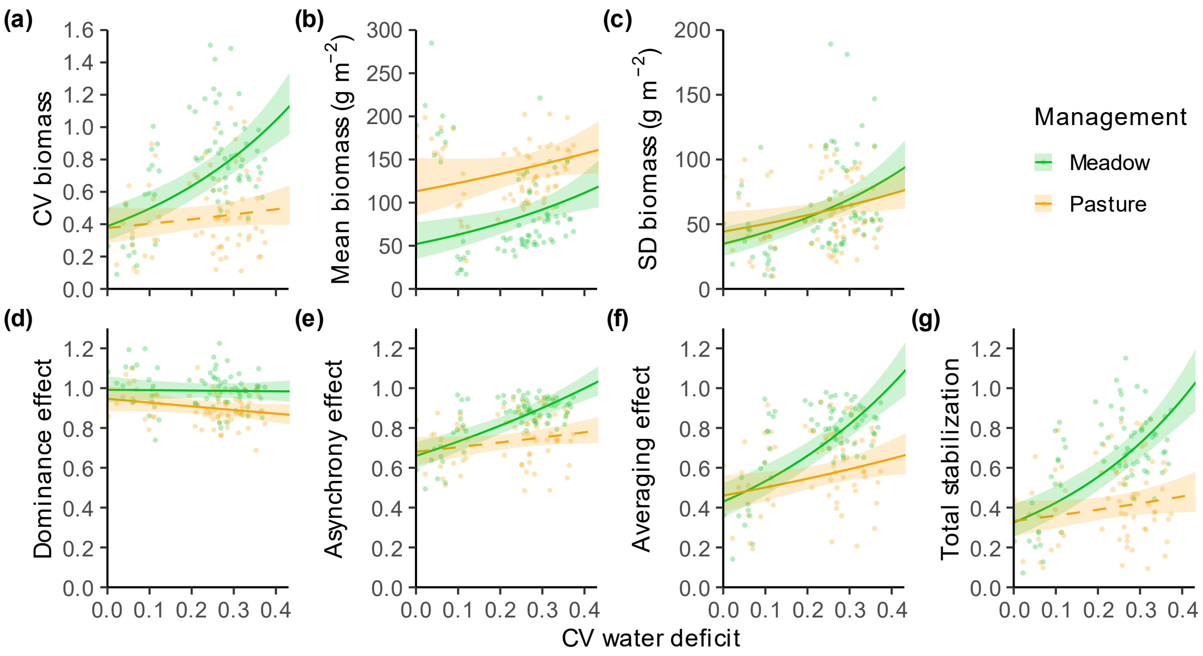

Fig. S10 Temporal (a) variability (CV), (b) mean, (c) standard deviation (SD), stabilization by (d) dominance, (e) asynchrony, (f) averaging, and (g) total stabilization of community biomass dependent on temporal variability (CV) of water deficit per grassland management treatment (across climate treatments, regression lines ± 95% CI, n = 70), calculated for seven overlapping time windows of three years (2015 – 2023) across summer harvests. Solid regression lines denote significant linear relationships (P < 0.05, Table S8). Background points show the raw data (climate treatments not distinguished). Lower values in (d-g) indicate higher stabilization, while values above one imply destabilization

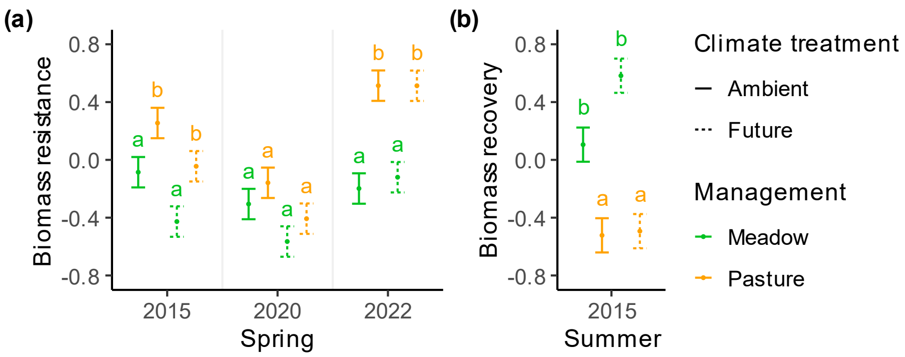

Fig. S11 Community biomass (a) resistance to and (b) recovery from extreme dry springs per climate × grassland management treatment combination (predicted means ± SE, n = 5). Recovery was assessed in the following summers, provided these were classified as normal in terms of water conditions, which was not the case in 2020 and 2022. Different lowercase letters denote significant differences ($P < 0.05$, Table S9)

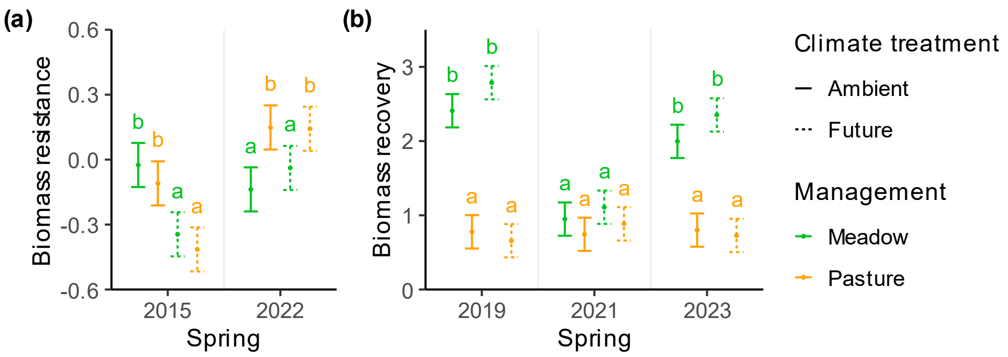

Fig. S12 Community biomass (a) resistance to extreme dry springs (2020 excluded due to different spring harvest dates in the management types) and (b) recovery from extreme dry summers per climate × grassland management treatment combination (predicted means ± SE, n = 5). Recovery was assessed in the following springs, provided these were classified as normal in terms of water conditions, which was not the case in 2020. Community biomass in springs was adjusted by adding biomass uptake during early spring grazing. Different lowercase letters denote significant differences ($P < 0.05$, Table S10)
